# Active hepatitis C infection and HCV genotypes prevalent among the IDUs of Khyber Pakhtunkhwa

**DOI:** 10.1186/1743-422X-8-327

**Published:** 2011-06-28

**Authors:** Latif ur Rehman, Ihasn Ullah, Ijaz Ali, Imtiaz Ali Khan, Aqib Iqbal, Sanaullah Khan, Sher Hayat Khan, Khaleeq Uz Zaman, Najib ullah Khan, Zahoor Ahmed Swati, Anila Tariq Jahangiri

**Affiliations:** 1 Institute of Biotechnology and Genetic Engineering, KPK Agricultural University, Peshawar, Pakistan; 2 Department of Entomology, KPK-Agricultural University, Peshawar, Pakistan; 3 Kohat University of Science and Technology, Kohat, Pakistan; 4 Department of Animal Health, KPK-Agricultural University, Peshawar, Pakistan

**Keywords:** IDUs, HCV, Genotype, RT-PCR, KPK

## Abstract

Injection drug users (IDUs) are considered as a high risk group to develop hepatitis C due to needle sharing. In this study we have examined 200 injection drug users from various regions of the Khyber Pakhtunkhwa province for the prevalence of active HCV infection and HCV genotypes by Immunochromatographic assays, RT-PCR and Type-specific PCR. Our results indicated that 24% of the IDUs were actively infected with HCV while anti HCV was detected among 31.5% cases. Prevalent HCV genotypes were HCV 2a, 3a, 4 and 1a. Majority of the IDUs were married and had attained primary or middle school education. 95% of the IDUs had a previous history of needle sharing. Our study indicates that the rate of active HCV infection among the IDUs is higher with comparatively more prevalence of the rarely found HCV types in KPK. The predominant mode of HCV transmission turned out to be needle sharing among the IDUs.

## Introduction

Hepatitis C is an infectious disease affecting the liver, caused by the hepatitis C virus [1]. Infection with HCV becomes persistent in > 70% of infected people and may be associated with chronic hepatitis, cirrhosis and hepatic cell carcinoma [2]. Approximately a quarter of a million deaths per annum occur due to chronic liver disease associated with HCV [3].

Hepatitis C continues to be a major disease burden on the world. According to the WHO estimates, 3% of the worldwide population is infected with the hepatitis C virus [4]. The prevalence of chronic hepatitis C in the Asia-pacific region is variable between 4% to 12% [5].

The overall observed modes of transmission in Pakistan are multiple use of needles/syringes (61.45%), major/minor surgery/dental procedures (10.62%), blood transfusion and blood products (4.26%), sharing razors during shaving or circumcision by barbers (3.90%), piercing instruments, nail clippers, tooth brushes, and in less than 1% due to needle stick, from infected mother to baby and sexual transmission [6,7].

A growing risk for the transmission of blood borne diseases in Pakistan is related to injection drug use [8]. In Indonesia, China, Vietnam, Eastern Europe and Central Asia outbreaks of HCV have been associated with injection drug use [8-12]. Pakistan is considered to be the main trafficking route for opiates from Afghanistan which produces the largest bulk of opium [13]. A recent report by the United Nations estimated a country-wide annual prevalence of 0.8% of opiate use in Pakistan [14]. Earlier studies have observed high HCV seroprevalence among the Injection drug users in two cities of Pakistan [15,16]. In Khyber Pakhtunkhwa province of Pakistan, IDUs have never been investigated for active HCV infection or prevalent HCV genotypes. As seroprevalence of anti-HCV does not tell us about whether the subjects are actively infected and there was lack of prevalence data on the active HCV infection or prevalent HCV genotypes, therefore we undertook the study to analyze the presence of HCV RNA and HCV genotypes prevalent among the IDUs belonging to various regions of Khyber Pakhtunkhwa province of Pakistan. The study also investigated the most common risk factors for the transmission of HCV among the IDUs.

## Materials and methods

### Sampling

The study included IDUs from various parts of Khyber Pakhtunkhwa including District Peshawar, District Mardan and District Kohat. A proforma was filled by each of the IDUs which contained information about previous history of needle sharing, major or dental surgery, blood transfusion, marital status and age etc. 5 ml of blood was collected in each case in separate disposable syringes and transported to the Institute of Biotechnology and Genetic Engineering, Peshawar where serum separation was carried out. The study had been approved by the board of study of IBGE. All experiments were performed in accordance with the ethical standards mentioned in the declaration of Helsinki.

### Immunochromatographic Tests (ICT)

Screening for HCV positive samples was carried out with the help of Immuno-chromatographic tests (Accurate, USA) followed by (Acon, USA). Samples positive by ICT were furthered for next step evaluation.

### RNA extraction and Qualitative PCR

HCV RNA was extracted from 100 μl serum by using Ana-gen RNA extraction kit (Ana-gen, USA) according to the manufacturer's instructions. Qualitative detection of serum HCV RNA was performed by Reverse transcription PCR as described previously [17].

### HCV Genotyping

Genotyping of HCV was done according to the previously mentioned Type-specific PCR method [18].

All The PCR products were analyzed on 2% agarose gel prepared in 0.5% TBE buffer, stained with ethedium bromide (10 μg/ml) as florescent dye. Gels were photographed using Alpha quant (Alpha Innotech). 100-bp DNA ladder (Gibco BRL) was used as DNA size marker.

## Results

**Figure 1:**
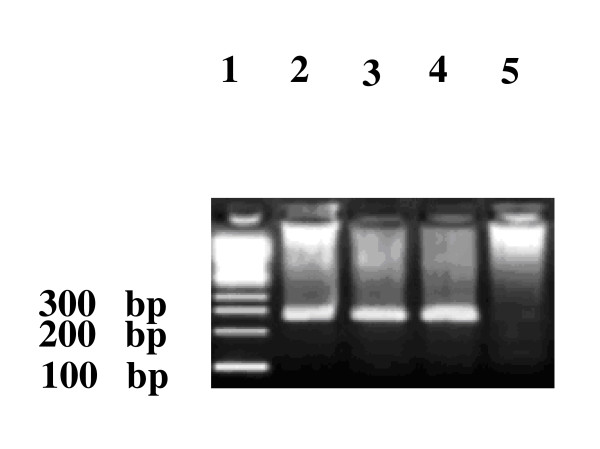
**Gel photograph of HCV PCR products: Lane 1 shows 100 bp DNA ladder, lane 2, and 3 are the positive samples for active HCV infection showing 285 bp nested PCR product, Lane 4 is positive HCV control while Lane 5 is negative control**.

It is evident from the previous studies conducted in Pakistan that Injection drug use is a predominant mode of HCV transmission. We analyzed the blood samples of 200 IDUs belonging to various districts of the Khyber Pakhtunkhwa province (Table 1) for the prevalence of active HCV infection and HCV genotypes. Our results indicated that out of the total 200 IDUs, 48 (24%) IDUs had active HCV infection as detected by RT-PCR (Figure 1). Comparatively high percentage (31.5%) of the IDUs had anti HCV in their blood (Table 2). Active HCV was more prevalent in district Peshawar followed by district Mardan and district Kohat (Table 2). Prevalent HCV genotypes were 2a (35.71%), 3a (28.57%), 4 (14.29%) and 1a (7.14%) while the genotypes in 4 (14.29%) IDUs could not be determined by the assay performed in this study (Table 3). Majority of the IDUs were married, economically very poor with an income of less than a dollar/day and had primary or middle school level education (Table 1). Needle sharing was observed in 95% cases.

**Table 1:** Demographics of IDUs

District	No. of IDUs	Male	Female	Married	Education	Economic status
Peshawar	100	98	2	62		
		
Kohat	60	60	-	30	Basic: 98%	< 1$/day: 95%
		
Mardan	40	40	-	28	High: 2%	Well off: 5%
		
Total	200	198	2	120		

**Table 2:** Prevalence of Anti-HCV and Active HCV infection among the IDUs

District	Total No. of IDUs	ICT positive	PCR positive
**Peshawar**	100	35 (35%)	30 (30%)

**Kohat**	60	15 (25%)	10 (16.67%)

**Mardan**	40	13 (32.5%)	8 (20%)

**Total**	200	63 (31.5%)	48 (24%)

**Table 3:** HCV genotypes prevalent among the IDUs of Khyber Pakhtunkhwa

HCV genotype	% prevalence
**2a**	35.71%

**3a**	28.57%

**4**	14.29%

**1a**	7.14%

**Untypeable**	14.29%

## Discussion

The burden of hepatitis C is increasing in Pakistan partly, due to lack of public awareness and poor screening facilities in our health care units. HCV is a blood-born pathogen and the investigated risk factors in Pakistan include major surgery, dental surgery, barbers, occupational needle pricks and Injection drug use [19-21]. Other studies from Pakistan have reported prevalence of anti-HCV antibodies among the IDUs from various parts of the country [15,16]. In this study, we investigated prevalence of active HCV infection among the IDUs and found that 24% of them had HCV RNA in their blood as detected by RT-PCR. The study revealed that a considerably higher percentage (31.5%) of the IDUs had anti-HCV antibodies in their sera (Table 2). Detection of more anti-HCV cases in this study could partly be attributed to the self limiting nature of the disease or the limitations of the immunochromatographic tests [22,23].

The distribution of HCV genotype 3 and 2 has been reported to be worldwide including Pakistan [17]. All the previous studies conducted in Pakistan have employed type specific PCR for the detection of various HCV genotypes. Earlier studies have reported that HCV genotype 3a is the most abundant genotype among the general population of Pakistan [17,24]. Our analysis indicated that genotype 2a was the most prevalent followed by 3a among the IDUs in Khyber Pakhtunkhwa province of Pakistan. HCV genotype 4 and 1a are only rarely found in Pakistan and have earlier been reported to be prevalent in Middle east, western countries, Australia and the Americas [25] but this study indicated that a considerable number of the IDUs were infected with genotype 4 (Table 3). Former history of the IDUs infected with genotype 4 and 1a revealed that a number of them had a history of residing in the Gulf region or Northerm America.

Injection drug use is uncommon among the female population of Khyber Pakhtunkhwa where social constraints do not allow free mix ups with the males and social interactions among the opposite sexes are limited. In our study, we analyzed 2 females from the entire province for HCV infection and both of them turned out to be negative for anti-HCV.

Demographics of the IDUs (Table 1) indicated that 60% of the IDUs were married. Married IDUs pose a greater risk of transmitting the disease to their spouses or siblings. Education status of 98% of the IDUs was basic (primary or middle school) and only 5% of the IDUs were economically well off. Due to poor economic status, increasing number of the IDUs have resorted to beggary causing serious social problems. We also noticed in this study that IDUs with a prolonged history of Injection drug use were more infected with HCV as compared to the novices. None of the IDUs had a history of major or dental surgery and only 5% had a history of blood transfusion. 95% of the IDUs had a previous history of needle sharing although some of them had quit with the practice. IDUs from Peshawar district were relatively more aware of the risk of needle sharing as compared to IDUs from other districts but non of them were informed about hepatitis C. Our study is in conformity with other studies conducted in Pakistan which had reported increasing trend of Injection drug use [26]. According to the results of National Assessment Study on Drug Abuse Situation in Pakistan, conducted in year 2000 it was estimated that about 60,000 drug addicts were using drugs through injections.

This study suggests that the government and non-governmental organizations should launch projects to educate people about hepatitis C and the transmission of HCV in order to minimize the eminent threat of the spread of the disease especially that of the rare genotypes which are comparatively less responsive to Interferon-based therapies. Policies regarding economic rehabilitation and psychological counseling for the war-affected people should help minimize the practice of Injection drug use.

## Conclusion

The study concludes that 24% of the IDUs in KPK province of Pakistan are actively infected with HCV. The prevalent HCV genotypes are 2a, 3a, 4 and 1a. Lack of awareness among the IDUs about needle sharing and increasing trend of Injection drug use due to regional socio-economic and geopolitical situation contributes a great deal towards the spread of HCV.

## Competing interests

The authors declare that they have no competing interests.

## Authors' contributions

IA designed the study and advised about the protocols. LR and IU carried out sampling and experimental procedures. IAK, ATJ, AI, SK, SHK, KUZ and NUK helped with experimental procedures and manuscript preparation. ZAS critically reviewed the manuscript. All authors read and approved the final manuscript.
